# Cardiac biomarkers for the quantification of myocardial damage after cardiac surgery – The RORSCHACH trial

**DOI:** 10.1016/j.ijcha.2025.101781

**Published:** 2025-08-28

**Authors:** Tulio Caldonazo, Marcus Winter, Michael Kiehntopf, René Aschenbach, Stephanie Gräger, Sebastian Reinartz, André Scherag, Ulrike Schumacher, Hristo Kirov, Ulf Teichgräber, Torsten Doenst, on behalf of the RORSCHACH Investigators and GermaN HeaRTS

**Affiliations:** a Department of Cardiothoracic Surgery, Friedrich-Schiller-University Jena, University Hospital Jena, Germany; b Center for Clinical Studies, University Hospital Jena, Germany; c Department of Clinical Chemistry and Laboratory Diagnostics, University Hospital Jena, Germany; d Department of Diagnostic and Interventional Radiology, University Hospital Jena, Germany; e Institute of Medical Statistics, Computer and Data Sciences, University Hospital Jena, Germany

**Keywords:** Cardiac biomarker, Myocardial infarction, Troponin

## Abstract

**Background:**

Cardiac biomarkers are important components for diagnosing perioperative myocardial infarction (MI). Efforts to detect MI by biomarker-release only faced heavy criticism, because cardiac biomarker-release has also been observed in situations that are not always related to cell death (e.g., renal insufficiency, neurological diseases, and even after endurance exercise). This study correlates release patterns of all three classically used cardiac injury biomarkers (CK/CK-MB, Troponin T and I) with myocardial damage visualized by late gadolinium enhanced cardiac magnetic resonance imaging (LGE-cMRI) and also compares biomarkers among each other.

**Methods and analysis:**

The RORSCHACH study is a prospective, multicenter, single-armed, non-blinded, non-controlled study evaluating cardiac biomarker release during elective aortic or mitral valve surgery and their correlation to perioperative myocardial damage as detected by MRI. Enrolled patients undergo routine monitoring including echocardiography, electrocardiography, cardiac biomarker analyses, and clinical symptom assessment preoperatively (within 24 h prior to surgery) and postoperative at predefined timepoints. LGE-cMRI is performed preoperatively and at least 5 days after surgery to clinically quantify any new myocardial damage. In total, 100 patients will be enrolled, whereby a drop-out rate of 15 % subsequently results in 85 patients necessary for final analysis. The primary endpoint is the correlation of the peak value of the respective biomarker with the amount of perioperatively induced myocardial damage quantified by LGE-cMRI.

**Discussion:**

The RORSCHACH trial will deliver the first comparative and quantitative information on the predictive value of the three classic cardiac injury markers used for the detection of new perioperative irreversible injury/MI in cardiac surgery.

**Study registration:**

Clinicaltrials.gov. NCT06066970. Registered on September 28th 2023.

## 1 Introduction

Cardiac biomarkers are important components for diagnosing perioperative myocardial infarction (MI). However, their reliability as sole diagnostic indicators remains controversial [1]. Elevated levels of cardiac biomarkers, such as troponin I (TnI), troponin T (TnT), creatine kinase (CK), and its MB isoenzyme (CK-MB), are not exclusively linked to ischemic events [2,3]. Non-ischemic conditions, including renal insufficiency, neurological disorders, and even physical exertion from endurance sports, have been associated with increased biomarker release [[2], [3], [4], [5]], which means that the elevation of biomarkers may not automatically be translated to the presence of irreversible cell injury.

The use of the biomarkers for defining periprocedural MI in current trials has therefore sparked considerable controversy [[6], [7], [8]]. The biomarker elevation can complicate the differentiation between true ischemic myocardial injury and other contributing factors, thereby emphasizing the need for more comprehensive diagnostic approaches. Currently, there are different types of evidence searching for a more precise correlation between biomarkers kinetics and possible myocardial damage in consensus [9], guideline [10] or universal definition level [11].

Recently, novel imaging techniques (e.g. late gadolinium enhancement cardiac magnetic resonance imaging − LGE-cMRI) showed to have the potential to detect myocardial damage through surrogate indicators, providing valuable insights into early pathological changes and facilitating timely diagnosis and intervention. In light of these limitations, this study aims to investigate the relationship between the perioperative release of cardiac biomarkers and detectable myocardial damage through LGE-cMRI.

## 2 Methods and analysis

### 2.1 Overview of the study design

**Fig. 1:**
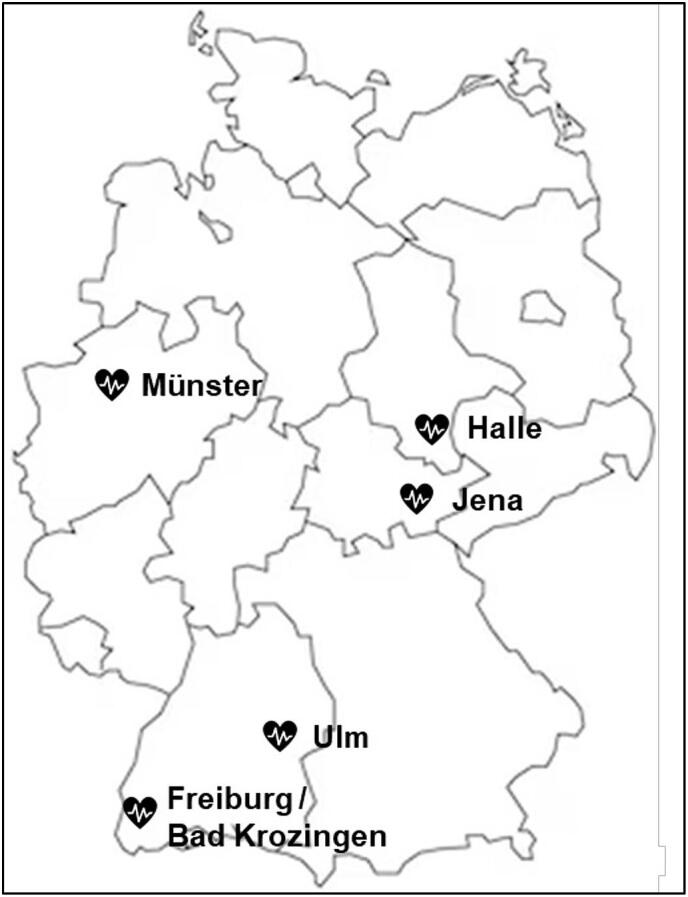
Participating heart centers in Germany.

The RORSCHACH study is an investigator-initiated, prospective, multicenter, single-armed, non-blinded, non-controlled study. Adult patients undergoing elective aortic or mitral valve surgery will be considered for study participation. Enrolled patients will receive routine monitoring including echocardiography, electrocardiography, cardiac biomarker analysis, clinical symptom assessment at defined timepoints. LGE-cMRI is performed preoperatively (within 24 h before surgery) and at least 5 days after surgery. Cardiac Biomarker analyses, namely TnI, TnT, CK, and CK-MB, as well as LGE-cMRI analyses will be performed centrally at a Core Lab in Jena. A total of 100 patients including drop-outs are planned to be included. There are eight different participating sites in Germany, of which five a currently initiated (Fig. 1). All sites provide the necessary staff and equipment with regard to conducting LGE-cMRI.

### 2.2 Objectives

#### 2.2.1 Primary objective

The primary objective of the study is to assess whether the perioperative releases of TnI, TnT, CK, and CK-MB correlate with myocardial damage, visualizable by LGE-cMRI. The primary endpoint is the correlation of the peak value of the respective biomarker with the amount of perioperatively induced myocardial damage quantified by LGE-cMRI.

#### 2.2.2 Secondary objective

This study will also address further endpoints and questions that, while not directly related to the primary study objective, are clinically highly relevant with regard to perioperative mortality and morbidity. These secondary endpoints comprise of the perioperative complications mentioned in section *Possible Complications and/or Risks*.

### 2.3 Study population

The study population comprises of adult patients undergoing elective aortic or mitral valve surgery. Eligible patients must not be diagnosed with coronary heart disease to minimize the probability of myocardial damage due to coronary pathology or coronary complications.

#### 2.3.1 Inclusion criteria

• Indication for aortic or mitral valve surgery
• Written informed consent
• Age ≥ 18 years

#### 2.3.2 Exclusion criteria

• Presence of coronary artery disease (excluded within the last 6 months)
• Allergy to gadolinium
• Non-MRI-competent (metal) implants (e.g. cardiac pacemaker, joint prothesis)
• Cochlear implant
• Deep brain stimulation
• Individual factors excluding the performance of an MRI (e.g. claustrophobia of the patient)
• Significantly reduced renal function (GFR < 30 ml/min)
• Perioperative complications that may lead to myocardial damage
• Need for extension of surgery
• Pregnancy or lactation

### 2.4 Obtaining informed consent

The nature of the study must be explained to each subject followed by written informed consent prior to enrollment. Obtaining written informed consent will then be carried out according to the Declaration of Helsinki and ICH-GCP E6. All patients enrolled must be capable of understanding the study design adequately and giving written informed consent by themselves. Study inclusion by means of a legal representative is not permitted.

### 2.5 Screening

Patient recruitment is carried out as part of the elective patient treatment. Patients are assigned on the basis of the indication for surgery (isolated aortic or mitral valve surgery). The exclusion of stenosing coronary heart disease by means of coronary angiography or coronary CT must have been performed preoperatively within the last 6 months. Since the RORSCHACH study is single-armed and non-blinded no randomization and blinding procedures are performed.

### 2.6 Frequency and scope of visits

**Fig. 2:**
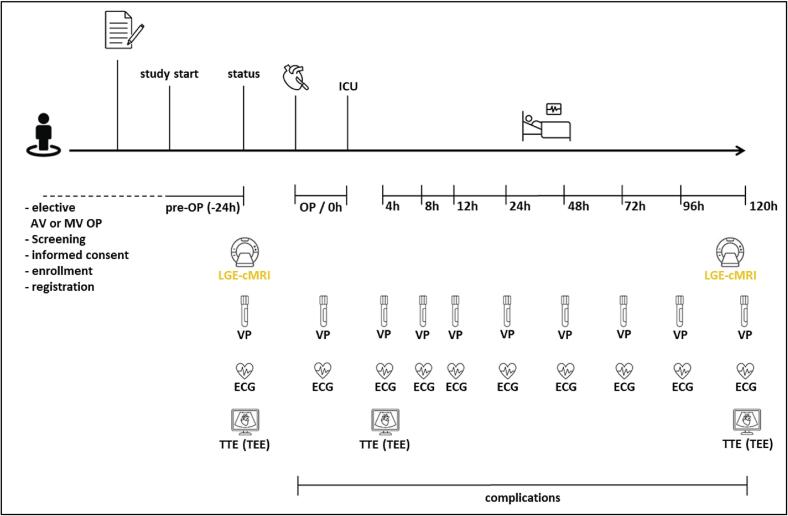
Study timeline. AV, aortic valve; ECG, electrocardiography; h, hour; ICU, intensive care unit, LGE-cMRI, late gadolinium enhancement cardiac magnetic resonance imaging; MV, mitral valve; OP, operation; TEE, transesophageal echocardiography; TTE, transthoracic echocardiography; VP, venipuncture.

Table 1 and Fig. 2 show the frequency and scope of trials’ visits. The study duration for the individual patient is at least 6 days comprising of a preoperative day and 5 days post surgery. After day 5 all diagnostics and sampling have been carried out and the patients’ end of study is reached. There will be no follow-up period.

**Table 1:** Frequency and scope of RORSCHACH visits.

Timepoint	Pre-OP	0 h	4 h	8 h	12 h	24 h	48 h	72 h	96 h	120 h
Medical history / baseline	X	-	-	-	-	-	-	-	-	-
Informed consent	X	-	-	-	-	-	-	-	-	-
Checking inclusion and exclusion criteria	X	-	-	-	-	-	-	-	-	-
Checking perioperative complications leading to myocardial damage (immediate end of study)		X	X	X	X	X	X	X	X	X
Physical examination	X									
LGE-cMRI	X	-	-	-	-	-	-	-	-	X
ECG	X	X	X	X	X	X	X	X	X	X
TTE (eventually TEE)	X	(-)	X	(-)	(-)	(-)	(-)	(-)	(-)	X
Sampling for central laboratory (TnI, TnT, CK, CK-MB)	X	X	X	X	X	X	X	X	X	X
Recording complications	-	X	X	X	X	X	X	X	X	X

(-): no TTE (eventually TEE) planned, only performed in case of possible myocardial damage. CK, creatine kinase; CK-MB, creatine kinase muscle-brain isoenzyme; ECG, electrocardiography; h, hour; LGE-cMRI, late gadolinium enhancement cardiac magnetic resonance imaging; OP, operation; TEE, transesophageal echocardiography; TnI, Troponin I; TnT, Troponin T; TTE, transthoracic echocardiography.

Enrolled patients undergoing isolated aortic or mitral valve surgery receive routine monitoring according to local routine including clinical symptom assessment, transthoracic echocardiography (TTE), 12-channel electrocardiography (ECG), and preoperative laboratory diagnostic. Transesophageal echocardiography (TEE) is only performed if medically indicated. Diverging from clinical routine patients undergo blood sampling for assessment of cardiac biomarkers (TnI, TnT, CK, CK-MB) as well as LGE-cMRI. Isolated aortic or mitral valve surgery is conducted according to local clinical standards.

Further diagnostic measures are done postoperatively as indicated above. Blood sampling for cardiac biomarkers and ECG are performed at patients’ arrival on intensive care unit (ICU) (0 h) as well as after 4 h, 8 h, 12 h, 24 h, 48 h, 72 h, 96 h, and 120 h. Echocardiography is done fast-track (4 h − analysis of left ventricular ejection fraction and possible wall motion abnormality) and in a detailed manner before discharge (120 h). Patients receive one additional LGE-cMRI after at least 120 h. From surgery to the patients’ individual end of study participation complications are monitored and documented continuously.

### 2.7 Blood sampling / sample processing / laboratory analysis of cardiac biomarkers

Blood sampling is performed during clinical routine, whenever possible via the venous central catheter, otherwise via venous puncture. Blood processing to gain study-specific serum and plasma samples are performed as described in the study laboratory manual based on the manufacturer’s instructions. Plasma and serum samples are stored at −80 °C freezers at the respective site. After a maximum storage time of 3 months all samples are shipped on dry ice to the Department of Clinical Chemistry and Laboratory Diagnostics (IKCL − Core Lab) in Jena via a professional laboratory courier service. Cardiac biomarker analysis of TnT, CK, and CK-MB in plasma samples are performed after arrival at the IKCL in Jena according to local routine. The time points of assessment will then allow, for the first time, comparing all three biomarkers and their dynamic and kinetic behaviors with each other. Cardiac biomarker analysis of TnI in serum samples are performed after arrival at the MVZ in Cottbus according to local routine.

### 2.8 LGE-cMRI / analysis

For conducting LGE-cMRI study-uniformly a MRI manual has been provided to all participating sites. Myocardial injury will be defined by the presence of abnormalities in T1/T2 mapping, extracellular volume, contractility and/or LGE. The process of mapping provides a quantitative means of objectifying alterations in the myocardium. The extent of left ventricular (LV) enlargement will be measured as a percentage of LV mass. The remaining qualities will be measured according to the AHA-Myocardial Segments classification system. To improve specificity, it will be performed complementary sequences such as T1/T2 mapping and extra cellular measurements to differentiate acute inflammatory changes from chronic scarring. During site initiation and prior to study-specific MRIs each site confirmed that MRI sequencing indicated in the manual is according to local clinical routine. Study-specific MRI data of all participating patients enrolled outside Jena will be transferred to Department of Diagnostic and Interventional Radiology (IDIR – Core Lab) in Jena for central analysis. Data transfer will be performed via TeamBeam, a data protection-compliant data exchange platform for location-independent, encrypted data exchange and for encrypted data storage comprising sensitive data, including email exchange with external partners. The data to be exchanged is password-protected, provided with a validity period, and is automatically deleted from the platform. The analyses will be performed in a blinded core lab (University of Jena) throughout one single investigator in order to reduce intra- and inter-observer variability.

### 2.9 Possible complications and/or risks

No additional complications and/or risks for the patients during study participation are expected. Based on the nature of the study and its regulatory classification no safety reporting is required. Nevertheless, for secondary endpoint assessment as well as due to possible study exclusion of patients after enrolment the focus will be on the documentation of the following events:

o death.
o MI with angiographic evidence of coronary ischemia.
o stroke.
o postoperative AV block II° or III° and need for pacemaker implantation
o re-thoracotomy due to bleeding and/or cardiac tamponade.
o postoperative delirium.
o respiratory insufficiency with need for re- or protracted invasive ventilation
o pneumonia.
o acute renal failure with indication for dialysis.
o allergic reaction to gadolinium (of any degree).
o perioperative complications resulting in study exclusion (coronary complications, extension of the operation beyond isolated heart valve operation, prolonged extracorporeal circulation e.g ECMO application, resuscitation).

### 2.10 Statistical considerations and methods

The statistical approach for the RORSCHACH study is exploratory and essentially descriptive. As mentioned previously, the primary endpoint is the correlation (Spearman's ρ) of the peak value of the respective biomarker (TnI, TnT, CK, CK-MB) with the amount of perioperatively induced myocardial damage quantified by LGE-cMRI. In addition, adequate methods of descriptive statistics to summarize biomarker distributions, exploratory bivariate correlation analyses (Spearman's ρ) and regression modeling (mixed, general linear models) including sensitivity analyses (e.g. stratified by study center) will be performed. There will be no adjustment for type I error.

### 2.11 Sample Size

With 85 evaluable patients, who are considered recruitable for practical reasons, α = 0.05 and a power = 0.8 for the primary endpoint H1: ρ = 0 against H0: ρ = 0.3 can be detected. Assuming a drop-out rate of 15 %, the required number of patients to be included is 100.

### 2.12 Interim analysis

There will be no interim analysis.

### 2.13 Methods against bias

All participating sites are certified cardiothoracic centers and have a quality management system. Investigators have to be trained in good clinical practice (GCP). All surgeons should be qualified cardiac surgeons and hold the appropriate national diploma or at least operate under surveillance of a senior cardiac surgeon. At each visit the occurrence of possible outcomes will be documented in the case report form (CRF).

Ultimately, patients who experience definitive intraoperative shock necessitating resuscitation doses or comparable interventions will be included in separated analyses. Intraoperative shock will be defined as any of: sustained hypotension (MAP < 40 mmHg ≥ 20 min), sustained high-dose vasopressors (norepinephrine ≥ 0.10 µg/kg/min, epinephrine ≥ 0.05 µg/kg/min, vasopressin ≥ 0.03 U/min, or ≥ 2 concurrent agents for ≥ 10 min), resuscitation interventions (CPR/defibrillation; push-dose vasopressor rescue ≥ 100 µg epinephrine or ≥ 400 µg phenylephrine within 10 min; massive transfusion ≥ 4 PRBC/60 min), or metabolic/oxygenation evidence of shock.

### 2.14 Ethical considerations

Approval for the study was obtained by the leading ethics committee of the Friedrich Schiller University Jena (no. 2022–2559-BO). In addition, and insofar necessary according to local regulations, the responsible ethics committees of participating sites approved the study protocol before conducting any study-specific measures.

### 2.15 Data management

Data acquisition will be done via web application into the study management software OpenClinica®. The software meets the regulatory requirements (GCP, 21CFR Part11). The data will be entered via encrypted connection (https) in web browser input masks. Each subject will be given an unambiguous patient identification number (subject ID) to ensure pseudonymized data analysis.

### 2.16 Privacy, collection and processing of data

The data obtained in the course of the study will be treated pursuant to the appropriate Data Protection Law. During the study, subjects will be identified solely by an individual patient identification number (subject ID). Study findings stored on a computer/server will be stored in accordance with local data protection law and will be handled in strictest confidence.

### 2.17 Study Status

The RORSCHACH study started recruiting in September 2023. Recruitment is planned to be completed by September 2025.

### 2.18 Dissemination

The results of this study will be published in a renowned international medical journal, irrespective of the outcomes of the study.

## 3 Discussion

The RORSCHACH trial will deliver the first comparative and quantitative information on the predictive value of the three classic cardiac injury markers used for the detection of new perioperative irreversible injury/MI in cardiac surgery.

Cardiac biomarkers are essential tools for diagnosing perioperative MI, yet their specificity remains contentious. We had discussed the inadequacy of defining MI solely based on biomarkers before [3]. A thorough comparison between different biomarkers after cardiac surgery and the quantitative relation to myocardial damage is lacking. The topic has raised substantial attention with several prominent publications [[6], [7], [8]] attempting to define cut-off values for the detection of MI by biomarker release only. While it is clear, that every true MI will cause the release of the proteins used for MI detection (specifically if it is a larger infarction), at low to intermediate amounts of biomarker release, it is not clear which pathophysiology is associated with the release observation. Since these intermediate values are most frequent, it is important to provide clarity, especially since MI definitions based on biomarker release only have been and still are used [[6], [7], [8]] for determining trial endpoints, in which surgery is compared to less invasive treatment modalities. Since biomarker release may also be associated with surgical stress, without being directly related to myocardial injury, this possibility may introduce a significant bias into the interpretation of trial results.

Although CAD patients were excluded, intraoperative factors such as severe hypotension or high-dose vasopressor use could lead to myocardial injury independent of the surgical procedure, which are residual confounders which can be hardly addressed quantitatively. For this reason, patients who develop clear cases of intraoperative shock requiring resuscitation doses or similar interventions will be subject to separated analyses.

With RORSCHACH, we are going to deliver the first comparative and qualitative information on the predictive value of the classic cardiac biomarkers for the detection of new perioperative irreversible injury/MI in the heart surgery setting. Demonstrating this laboratory and radiological correlation would be one of the first evidence-based steps forward in optimizing the diagnosis of truly significant clinical events in patients after cardiac surgery. This trial will generate important information with significant implications for not only daily practice but importantly for the design of new clinical trials involving coronary artery disease.

## 4 RORSCHACH Investigators

Tulio Caldonazo, Marcus Winter, Michael Kiehntopf, René Aschenbach, Stephanie Gräger, Sebastian Reinartz, André Scherag, Ulrike Schumacher, Hristo Kirov, Ulf Teichgräber, Torsten Doenst, Ulrich Schneider, Philine Fleckenstein, Katrin Schwope, Murat Mukharyamov, Cora Richert, Andreas Hoffmeier, Vladyslava Stasii, Ali Yilmaz, Michael Bietenbeck, Tim Berger, Christopher Schlett, Christopher Schuppert, Gábor Szabó, Gábor Veres, Viktor Bánhegyi, Andreas Liebold, André Peres, Meinrad Beer, Steffen Klömpken.

## 5 Collaborators

MVZ Gemeinschaftslabor Cottbus GmbH, Cottbus, Germany. University Hospital Jena, Institute for Clinical Chemistry and Laboratory Medicine, Institute for Diagnostic and Interventional Radiology, Center for Clinical Studies. Participating German Sites (as it stands): University Hospital Münster, Clinic for Heart and Thoracic Surgery, Clinic for Cardiology I − Section for Heart Imaging. University Heart Center Freiburg-Bad Krozingen, Department of Cardiovascular Surgery. University Hospital Freiburg, Clinic for Diagnostic and Interventional Radiology. University Hospital Halle (Saale), Clinic and Policlinics for Heart and Thoracic Surgery, Clinic for Diagnostic and Interventional Radiology. University Hospital Ulm, Clinic for Heart and Thoracic Surgery, Clinic for Diagnostic and Interventional Radiology.

## 6 Contributors

TC is the principal Investigator. TC, MK and TD wrote the manuscript. AS was involved in the design and sample size considerations. US is the responsible biometrician. MK, AS, UT and TD are scientific advisors who participated in reviewing the manuscript. RA, SG and SR are responsible for the LGE-cMRT evaluations in the Core Lab. MK and CR are responsible for the biomarkers’ quantifications in the Core Lab. MW is the project manager. TC, HK, PF, KS and MM are responsible for patients’ recruitment at the University Hospital Jena. All coauthors read and proved the final manuscript.

## 7 Patient and public Involvement

Patients and/or the public were not involved in the design, or conduct, or reporting, or dissemination plans of this research.

## 8 Patient consent for Publication

Not required.

## CRediT authorship contribution statement

**Tulio Caldonazo:** Writing – review & editing, Writing – original draft, Visualization, Validation, Supervision, Software, Resources, Project administration, Methodology, Investigation, Funding acquisition, Formal analysis, Data curation, Conceptualization. **Marcus Winter:** Writing – original draft, Supervision, Data curation, Conceptualization. **Michael Kiehntopf:** Visualization, Validation, Software, Project administration, Conceptualization. **René Aschenbach:** Methodology, Formal analysis. **Stephanie Gräger:** Methodology. **Sebastian Reinartz:** Methodology, Conceptualization. **André Scherag:** Resources, Project administration, Methodology. **Ulrike Schumacher:** Methodology, Investigation, Formal analysis. **Hristo Kirov:** Writing – review & editing, Writing – original draft, Formal analysis, Data curation, Conceptualization. **Ulf Teichgräber:** Project administration, Investigation. **Torsten Doenst:** Writing – review & editing, Writing – original draft, Visualization, Validation, Supervision, Software, Resources, Project administration, Methodology, Investigation, Funding acquisition, Formal analysis, Data curation, Conceptualization.

## Funding

This study is funded partly by the Deutsche Herzstiftung (DHS, German Heart Foundation) and the Interdisziplinäres Zentrum für Klinische Forschung (IZKF, Interdisciplinary Center for Clinical Research, University Hospital Jena, Germany). TC was also funded by the Deutsche Forschungsgemeinschaft (DFG, German Research Foundation) Advanced Clinician Scientist Program OrganAge funding number 413668513, by the DHS funding number S/03/23 and by the IZKF. SG was funded by Clinician scientist Program (CSP-18) by Interdisciplinary Center of Clinical Research.

## Declaration of competing interest

The authors declare that they have no known competing financial interests or personal relationships that could have appeared to influence the work reported in this paper.

## Data Availability

The data underlying this article will be kept digital and on file for the next 10 years.
